# Intermittent Hematuria and Shock due to Uretero-Arterial Fistula Diagnosed by Provocative Angiography

**DOI:** 10.3400/avd.cr.26-00104

**Published:** 2026-09-18

**Authors:** Ren Saito, Yujiro Miura, Naoki Edo, Takuya Matsushiro, Shinichi Imai, Junki Shibata, Tomohiro Matsumoto, Takuji Yamagami, Naoya Ichimura, Rie Yoshimura, Kaya Atagi, Hiroto Osakabe, Satoshi Fukata, Keiji Inoue

**Affiliations:** 1 Department of Cardiovascular Surgery, Kochi Medical Hospital, Nankoku, Kochi, Japan; 2 Department of Diagnostic and Interventional Radiology, Kochi Medical Hospital, Nankoku, Kochi, Japan; 3 Department of Urology, Kochi Medical Hospital, Nankoku, Kochi, Japan

**Keywords:** uretero-arterial fistula, provocative angiography, endovascular treatment

## Abstract

Uretero-arterial fistula is a rare but life-threatening cause of massive hematuria. A 78-year-old woman with a history of pelvic surgery, radiation therapy, ileal conduit diversion, and long-term ureteral stenting presented with gross hematuria and anemia. Contrast-enhanced computed tomography (CT) and repeated angiography failed to identify the bleeding source. During recurrent hemorrhage, provocative angiography with negative-pressure aspiration through a ureteral catheter demonstrated extravasation from the right common iliac artery. Endovascular stent-graft repair achieved immediate hemostasis. This case highlights the diagnostic value of provocative angiography in challenging cases of uretero-arterial fistula.

## Introduction

Uretero-arterial fistula (UAF) is a rare but serious condition that can cause fatal massive hematuria. The major risk factors include pelvic surgery, pelvic radiotherapy, and long-term ureteral stenting. In patients with these backgrounds, fistula formation is thought to result from close contact between the ureter and the adjacent artery, together with chronic inflammation, fibrosis, and repetitive arterial pulsatile stimulation.

However, UAF is often difficult to diagnose because bleeding is intermittent and the fistula is frequently small, making localization by contrast-enhanced computed tomography (CT) or conventional angiography challenging. Delayed diagnosis may be fatal. Endovascular treatment is particularly useful in patients for whom open surgery is difficult because it enables minimally invasive and rapid hemostasis.

Here, we report a case of UAF in which localization of the fistula was difficult with conventional imaging, but the lesion was successfully diagnosed by provocative angiography combined with urinary tract manipulation and treated with endovascular therapy, resulting in a lifesaving outcome.

## Case Report

The patient was a 78-year-old woman. Four years earlier, she had undergone chemoradiotherapy for stage IVA cervical cancer, followed by total cystohysterectomy and ileal conduit urinary diversion. Thereafter, long-term bilateral ureteral stents were placed.

She was emergently admitted to the Department of Urology at our hospital because of progressive anemia and gross hematuria. After admission, she received blood transfusion and antibiotic therapy. On the day after admission, the bilateral ureteral stents were exchanged. Urine from the left ureteral stent was clear, whereas gross hematuria was observed from the right ureteral stent, raising suspicion of a right-sided culprit lesion. Laboratory findings on admission showed anemia, with a hemoglobin level of 6.0 g/dL, and renal dysfunction, with a serum creatinine level of 2.22 mg/dL. Her body temperature was 36.0°C on admission, but fever of up to 39.1°C developed on the night of admission. The white blood cell count and C-reactive protein (CRP) level were 24900/μL and 5.78 mg/dL, respectively. Urine culture was positive for Serratia species and methicillin-sensitive Staphylococcus aureus, whereas blood culture was negative. Pyelonephritis was suspected, and ceftriaxone (1 g twice daily) was initiated.

**Fig. 1:**
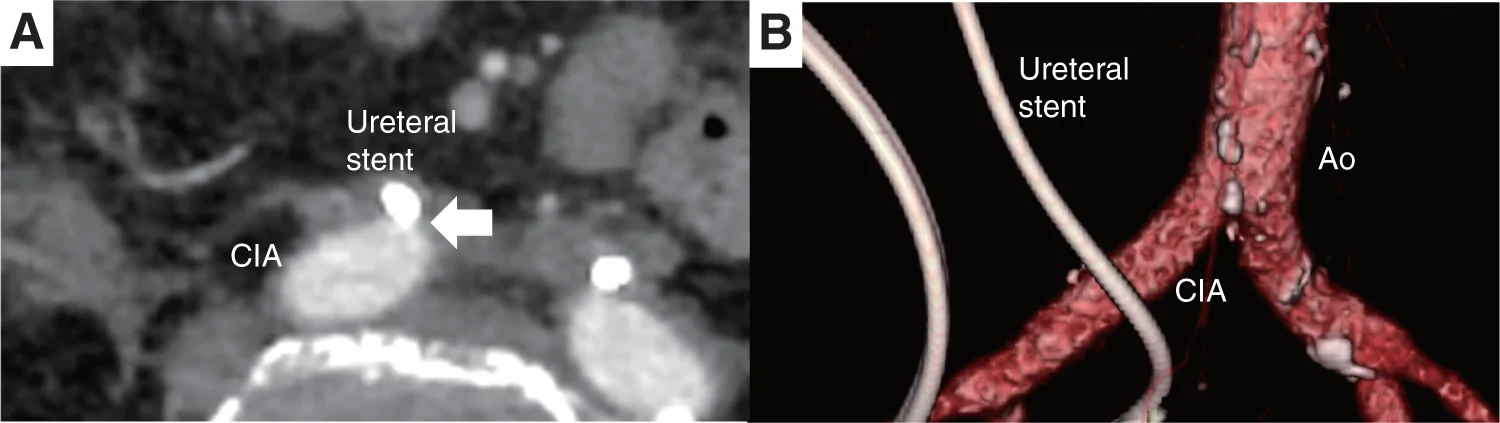
Contrast-enhanced CT findings suggestive of uretero-arterial fistula. (**A**) Axial CT image demonstrating close proximity between the right ureteral stent and the right CIA at the crossing point. (**B**) Three-dimensional reconstructed CT image showing the spatial relationship between the right ureteral stent and the right CIA. Ao, aorta; CIA, common iliac artery; CT, computed tomography

Contrast-enhanced CT showed no definite extravasation, intraluminal ureteral hematoma, or perilesional hematoma. However, the right common iliac artery (CIA) was in close proximity to the right ureteral stent (**Fig. 1**), whereas the left ureteral stent appeared more distant from the iliac artery and was separated by an intervening tissue plane. No ureteroscopic or ileal conduit endoscopic evaluation was performed. Based on these imaging findings together with her clinical background, UAF was suspected, and she was referred to our department. Emergency angiography was performed using a 5-Fr sheath, a pigtail catheter, and a 0.035-inch guidewire; however, no definite fistula or extravasation was identified. Because the hematuria subsequently showed signs of improvement, she was managed conservatively with close observation.

**Fig. 2:**
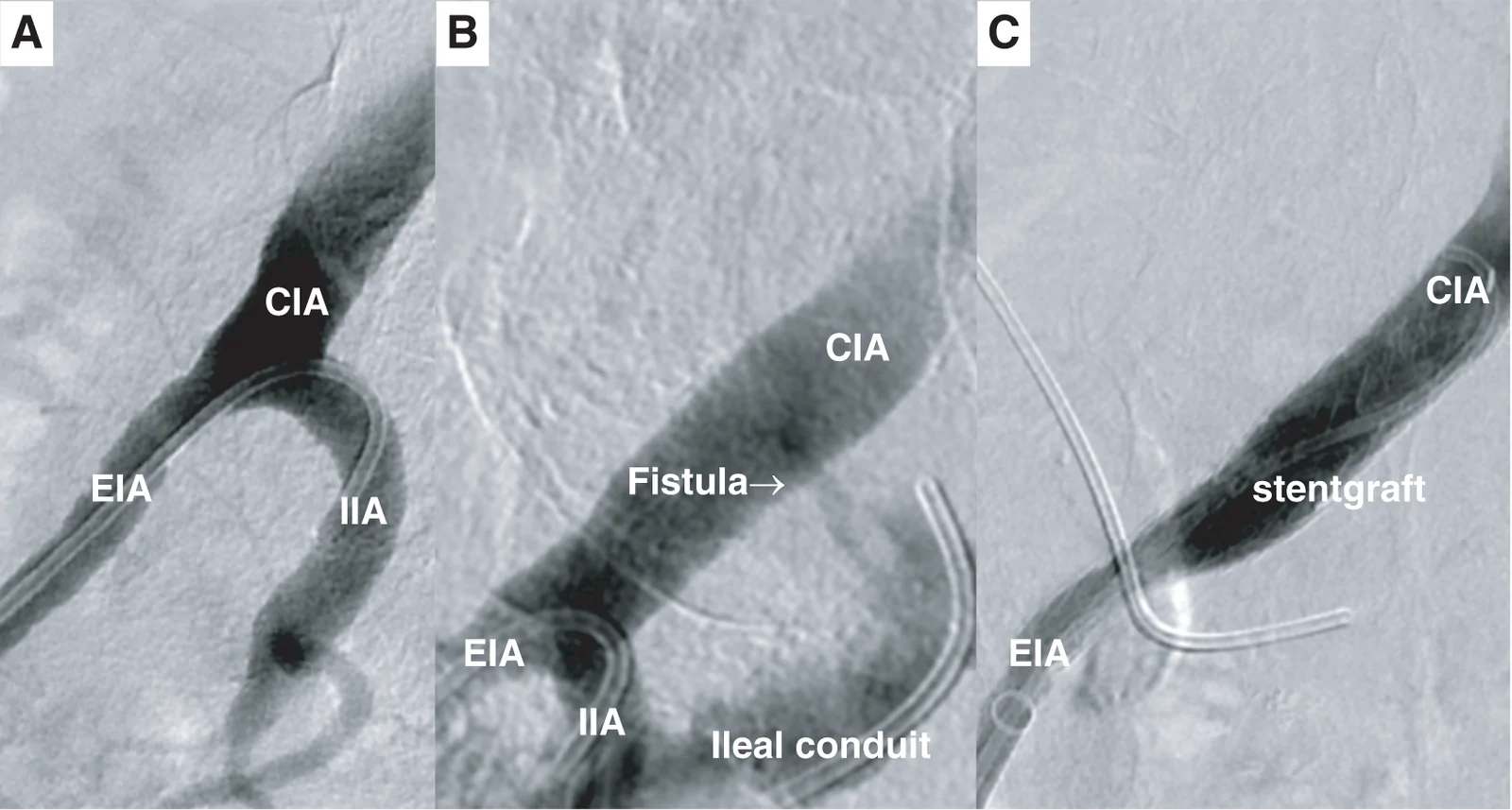
Intraoperative angiographic findings and endovascular treatment in chronological order. (**A**) Initial angiography without definite extravasation. (**B**) Provocative angiography under manual negative pressure through a ureteral catheter positioned near the crossing point, demonstrating extravasation from the right CIA. (**C**) Completion angiography after stent-graft deployment from the right CIA to the EIA, showing exclusion of the fistula, no residual fistulous opacification, no endoleak, no contrast extravasation, and no opacification of the IIA. CIA, common iliac artery; EIA, external iliac artery; IIA, internal iliac artery

On the following day, hematuria recurred, and she developed hemorrhagic shock due to massive bleeding. During transfer in hemorrhagic shock, manual compression was attempted, and the ureteral stent became unintentionally dislodged; therefore, it was not visible on the subsequent angiographic images. Repeat angiography was performed; however, as with the initial examination, the fistula site could not be clearly identified. Because the bleeding was intermittent, we suspected that the culprit lesion would be difficult to detect by conventional angiography alone. A new 5.0-Fr, 70-cm open-end ureteral catheter (Open-End Ureteral Catheter; Cook Medical, Bloomington, IN, USA) was then inserted through the ileal conduit into the right ureter, with the tip positioned near the crossing point between the ureter and the right CIA. Selective iliac arteriography was repeated while manual negative pressure was applied through the ureteral catheter using a syringe. Suction was applied smoothly without notable resistance, and this provocative maneuver demonstrated extravasation from the right CIA, leading to the diagnosis of a right UAF (**Fig. 2**). The procedure was performed under local anesthesia with ongoing transfusion support. A urologist remained at the patient’s head side throughout the procedure to provide immediate urinary tract manipulation and medication support if needed.

After identification of the fistula site, the vascular access was prepared for endovascular treatment, the guidewire was exchanged for a stiff guidewire, and the sheath was upsized to a 12-Fr DrySeal Flex Introducer Sheath (W. L. Gore & Associates, Flagstaff, AZ, USA). The diameter of the right CIA was approximately 10 mm and that of the right external iliac artery (EIA) was approximately 7 mm. The proximal landing zone measured approximately 17 mm. Given this relatively short proximal landing zone, we considered a GORE EXCLUDER Iliac Extender to be more suitable than a VIABAHN device for obtaining a secure proximal seal. Therefore, a GORE EXCLUDER Iliac Extender (12 × 70 mm; W. L. Gore & Associates) was deployed from the right CIA to the EIA. Embolization of the internal iliac artery (IIA) was not performed. No fistulous involvement of the IIA or aneurysmal change of the CIA was identified, and we considered the risk of retrograde type II endoleak to be low given this anatomy. Completion angiography demonstrated exclusion of the fistula, with no residual fistulous opacification, no endoleak, and no contrast extravasation. No opacification of the IIA was observed after stent-graft deployment.

Ceftriaxone had been administered for 6 days before the intervention, and the inflammatory markers improved to a white blood cell count of 11800/μL and a CRP level of 3.19 mg/dL immediately before the procedure. After the procedure, antibiotics were changed to cefepime and later to oral suppressive therapy; this was subsequently changed to minocycline according to microbiological findings and susceptibility. During 2 months of follow-up, no recurrent hematuria, stent-graft occlusion, pelvic ischemic symptoms, or clinical or laboratory worsening of infection were observed.

## Discussion

Although UAF is a relatively rare disease, it can lead to massive hematuria and hemorrhagic shock, and missed or delayed diagnosis may be fatal. In particular, a history of pelvic surgery, radiation therapy, and long-term ureteral stenting represents a typical high-risk combination, all of which were present in our patient.^1–4)^

The main reasons why UAF is difficult to diagnose are that the bleeding is intermittent, the fistula is often very small, and active bleeding may not be evident at the time of examination. Reported diagnostic sensitivities vary across reviews, but CT and CT angiography (CTA) generally has limited sensitivity, whereas angiography provides a higher yield. In a systematic review of 445 patients, CTA and angiography showed sensitivities of 48% and 62%, respectively. Another review reported diagnostic rates of 22.3% for CT and 46.8% for angiography. Therefore, UAF should not be excluded in patients with a highly suggestive clinical background, even when routine imaging is negative.^5,6)^

**Fig. 3:**
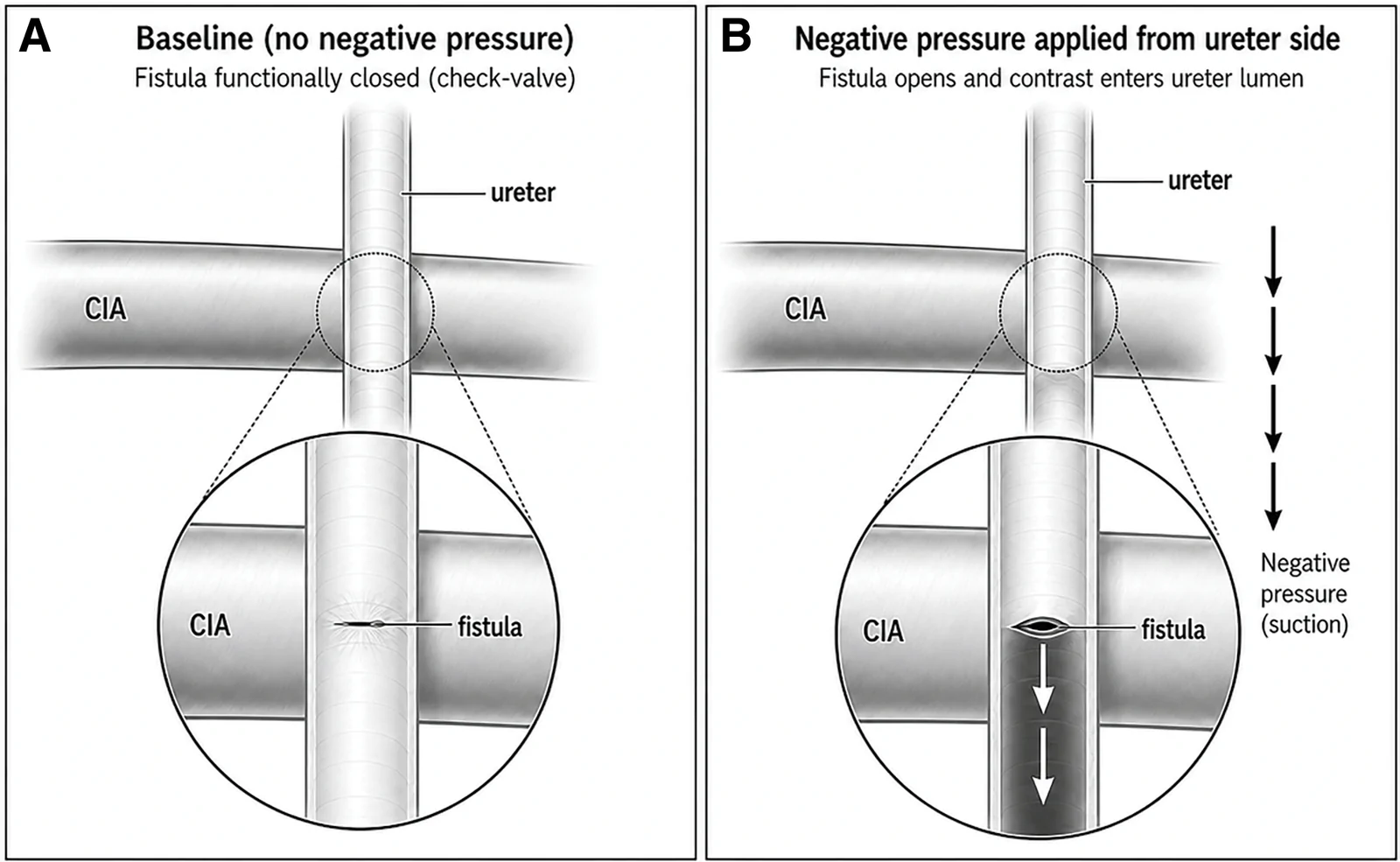
Schematic illustration of the presumed mechanism of provocative angiography in this case. (**A**) A tiny fistulous tract at the crossing of the right CIA and the ureter remains functionally closed, with no visible ureteral opacification on routine angiography. (**B**) Negative pressure applied through the ureteral catheter from the ileal conduit side opens the minute fistula and results in opacification of the ureteral lumen. Downward arrows indicate suction from the ureteral side. CIA, common iliac artery

In this case, the key to diagnosis was provocative angiography combined with urinary tract manipulation. Previously reported maneuvers include removal or manipulation of an indwelling ureteral stent, manipulation of a vascular catheter at the suspected crossing point, and balloon- or catheter-directed endoureteral mechanical friction. In a review of patients with ileal conduit urinary diversion, provoked angiography was diagnostic in 13 of 14 cases (92.9%), although these data should be interpreted cautiously because provocative maneuvers are not standardized. In our case, manual negative-pressure aspiration through the ureteral catheter functioned as a urinary-side provocative maneuver and enabled visualization of the fistulous communication.^7,8)^ Because provocative angiography can precipitate massive hemorrhage, it should be reserved for selected cases with strong clinical suspicion and performed only under controlled circumstances with immediate bailout capability. Suggested protective strategies include performing the procedure in an endovascular setting, preparing for immediate definitive treatment, and considering balloon occlusion when feasible. In our case, diagnosis was achieved without catastrophic deterioration under ongoing transfusion support, but this experience highlighted the importance of peri-procedural preparation.^5,8,9)^ The intermittent nature of hematuria in UAF is likely multifactorial. In addition to the speculative “check-valve” phenomenon (**Fig. 3**), temporary tamponade by an indwelling ureteral stent and transient occlusion of the fistulous tract by blood clot may also contribute. Changes in local hemodynamic conditions may further influence whether active extravasation is visible at a given time. Prior reports have similarly noted that UAF often presents with intermittent gross hematuria, which may lead to initially negative imaging studies.^10,11)^

With regard to treatment, open surgical revascularization or bypass surgery has traditionally been selected. However, in patients such as ours, who have a history of pelvic surgery, radiation therapy, and urinary diversion, surgical treatment is highly invasive because of local adhesions and the patient’s overall condition. In this context, endovascular treatment is minimally invasive and allows rapid hemostasis, making it an important therapeutic option, particularly in emergency cases complicated by hemorrhagic shock.

Delayed complications such as stent-graft infection, recurrent fistula formation, recurrent bleeding, and stent-graft occlusion should be recognized. In the review by Matsunaga et al., stent-graft infection was reported in 14.3% of endovascularly treated cases, and close follow-up during the first postoperative year was recommended. Although our follow-up is limited to 2 months, no recurrent hematuria, stent-graft occlusion, pelvic ischemic symptoms, or worsening infection has been observed.^7,12)^

## Conclusion

We experienced a case of UAF in which identification of the culprit lesion was difficult with conventional contrast-enhanced CT and angiography. UAF cannot be excluded even when imaging findings are negative, and a high index of clinical suspicion is essential. In selected cases, provocative angiography with careful safety planning may be useful when UAF is strongly suspected despite negative conventional imaging findings.
